# Supplementary material for: Representation of visual uncertainty through neural gain variability
Source: Nat Commun. 2020 May 19;11:2513. doi: 10.1038/s41467-020-15533-0 (PMC7237668; doi:10.1038/s41467-020-15533-0)
Supplement: Supplementary file 1 — Supplementary Information [file 41467_2020_15533_MOESM1_ESM.pdf]

Supplementary Information

Representation of visual uncertainty through neural gain variability

Olivier J. Hénaff, Zoe M. Boundy-Singer, Kristof Meding, Corey M. Ziemba, Robbe L. T. Goris

Different measures of neural response dispersion can behave differently

In the main paper, we compared two measures of neural response dispersion: gain variability and Fano factor (defined as the ratio of the spike count variance to the mean). We found the former to track stimulus uncertainty much better than the latter (see Fig. 3d and 4f). Why might this be so? In our experiments, the more uncertain stimulus conditions are associated with reduced responsiveness and increased gain variability. Under the modulated Poisson model, these effects act on the Fano factor in opposite manners. Everything else being equal, a decrease in responsivity will reduce Fano factor, while an increase in gain variability will increase Fano Factor (Supplementary Figure 1).

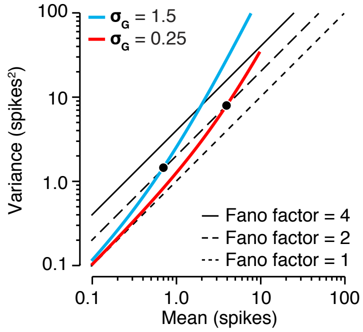

Supplementary Figure 1 Fano factor and gain variability can behave differently. Variance-to-mean relation under the modulated Poisson model is shown for two levels of gain variability (blue and red line). The black lines with unit slope each illustrate a constant value of Fano factor (set to 1, 2, and 4, respectively). When increases in gain variability are accompanied by a reduction in mean response, Fano factor can remain constant, as illustrated by the black points. This explains why Fano factor can be detached from stimulus uncertainty, even though gain variability is strongly associated with stimulus uncertainty (Fig. 3d; Fig. 4f).

Alternative comparison of models with slow and fast dynamics

In the main paper, we compared models with slow and fast dynamics by jointly fitting spikes binned in five different counting windows. While this analysis provided a clear result (see Fig. 6), its statistical soundness can be questioned because the same data are included multiple times to identify the models' parameters. As an alternative, we also performed an analysis in which we compared models with slow and fast dynamics by only fitting spikes binned using the longest counting window (1,000 ms). We then generated predictions for the spike count distributions in all other bin-sizes assuming either slow or fast dynamics (Supplementary figure 2a). The slow-dynamics model generated better predictions than the fast dynamics model in 88.85% of cases (Supplementary figure 2b). Thus, this alternative analysis supports the same conclusion as the analysis in the main text: gain fluctuations are better described as arising from a process with slow rather than fast dynamics.

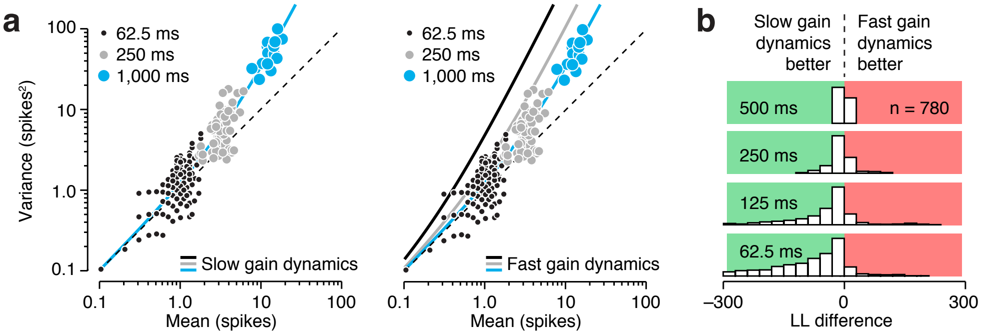

Supplementary Figure 2 Comparison of models with slow and fast gain dynamics. (**a**) Variance-to-mean relation for one stimulus family for the example V1 neuron from Fig. 6. Responses are shown for three different counting windows: 62.5 ms (black points), 250 ms (grey points), and 1,000 ms (blue points). We fit two models to the largest counting window data: one with fast gain dynamics (left panel), and one with slow gain dynamics (right panel). (**c**) We measured goodness-of-fit for the hold-out data by computing the log likelihood of the data at each window size for both models. Distribution of the difference in log likelihood under both models for a population of V1 and V2 neurons is shown for the 500 ms (top) to the 62.5 ms (bottom) counting window.
